# A sixfold rise in concurrent day and night-time heatwaves in India under 2 °C warming

**DOI:** 10.1038/s41598-018-35348-w

**Published:** 2018-11-16

**Authors:** Sourav Mukherjee, Vimal Mishra

**Affiliations:** Civil Engineering, Indian Institute of Technology Gandhinagar, Gujarat, 382355 India

## Abstract

Heatwaves with severe impacts have increased and projected to become more frequent under warming climate in India. Concurrent day and nighttime heatwaves can exacerbate human discomfort causing high morbidity and mortality; however, their changes in the observed and projected climate remain unrecognized. Here using observations and model simulations from climate of 20^th^ century plus (C20C+) detection and attribution (D&A) and coupled model intercomparison project 5 (CMIP5) projects, we show that 1 and 3-day concurrent hot day and hot night (CHDHN) events have significantly increased during the observed climate in India. Our results show that the anthropogenic emissions contribute considerably to the increase of 1 and 3-day CHDHN events in India. The frequency of 3-day CHDHN events is projected to increase 12-fold of the current level by the end of 21^st^ century and 4-fold by the mid 21^st^ century under the high emission pathway of RCP 8.5. The increase in 3-day CHDHN events can be limited to only 2-fold by the end of 21^st^ century under low emission scenario of RCP 2.6. One and 3-day CHDHN events are projected to increase by 4, 6, and 8 folds of the current level in India under the 1.5, 2, and 3 °C warming worlds, respectively. Restricting global mean temperature below 1.5° from the pre-industrial level can substantially reduce the risk of 1 and 3-day CHDHN events and associated implications in India.

## Introduction

An increase in the frequency and intensity of severe heat events due to anthropogenic emissions has been observed across the globe^1–5^. India has been substantially affected by deadly heatwaves in the past due to the warming climate and increasing population^5–11^. For instance, heatwaves during 1998 and 2015 caused more than 2000 deaths each in India. More importantly, exposure to the severe heatwaves^5^, heat stress^6,7^, and heat-related mortality^8^ in India are projected to increase significantly in the future.

Indicators to quantify the risks of heatwave vary and most of them are based on daily maximum temperature^9,10,12^ Health risks due to daytime heatwave can substantially increase if human body does not get a break from the heat during night^13^. Cooler nighttime temperature provides comfort from the daytime heat. However, if the stress originating due to daytime heatwave persists at night, it further exacerbates human discomfort and pre-existing health disorders^14–16^. Therefore, hot day and the hot night events occurring in the same calendar day may have significant implications on heat-related morbidity and mortality as observed for the 1995 Chicago heatwave^17^. An approach that integrates hot day and hot night events occurring on the same day (concurrent events) and persisting for more than 48-hours^17^ is necessary to adequately assess the impact of severe heat events under the current and future climate. Despite the implications of concurrent heat events, previous studies have either considered daily maximum (day-time), minimum (night-time), or average air temperature^5,8,18,19^ to quantify heatwave characteristics over India and ignored concurrent heatwaves.

Here, we provide a first-ever assessment based on concurrent hot day and hot night (CHDHN) events over India for the observed (1951–2016) and projected future climate. Apart from 1-day CHDHN events, we evaluate the changes in 3-day CHDHN events using 3-day moving average of daily maximum and minimum temperatures^17,20^. The effect of anthropogenic warming on CHDHN events is examined using simulations from Climate of 20^th^ century plus (C20C+) detection and attribution (D&A) project for the Historical (Hist) and Historical Natural (HistNat) scenarios. We report the changes in the frequency of CHDHN events in the future climate scenario using data from global climate models (GCMs) that participated in the Coupled Model Intercomparison Project Phase 5 (CMIP5). Finally, we report the changes in CHDHN events in India under the 1.5 °C, 2 °C, and 3 °C warming worlds.

## Results and Discussion

**Figure 1:**
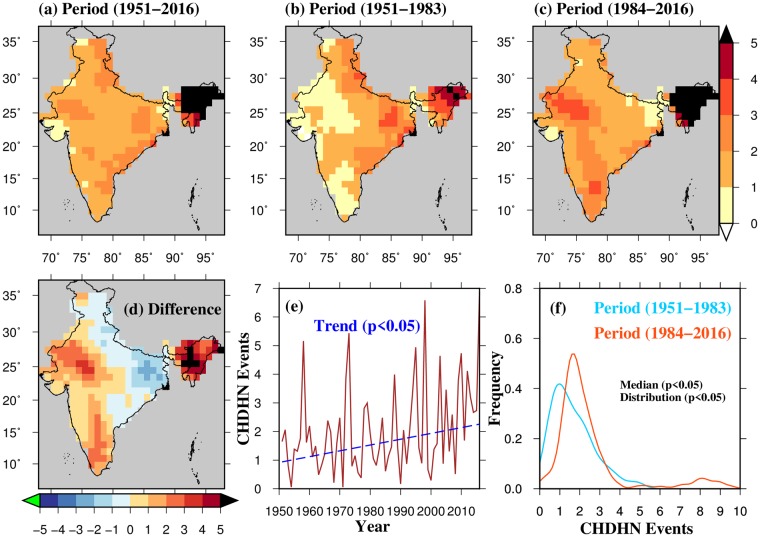
Changes in observed concurrent hot day and hot night (CHDHN) events. (**a**) Average frequency of 3-day CHDHN events for the period (1951–2016), (**b**) same as in (**a**) but for the period 1951–1983, (**c**) same as in (**a**) but for the period 1984–2016, (**d**) difference of (**b** and **c**), (**e**) all-India averaged frequency of 3-day CHDHN events (1951–2016), (**e**) kernel density functions for (**b**), and (**c**) and their kolmogorov-smirnov, and rank-sum significance test. The figure was developed using the Generic Mapping Tools (GMT, https://www.soest.hawaii.edu/gmt/).

First, we estimate the average frequency of 3-day CHDHN events during the observed period of 1951–2016 (Fig. 1). On an average two 3-day CHDHN events per year occurred over most of India during 1951–2016 suggesting that 3-day CHDHN are not common during the summer (April-June) season in the observed climate. To analyze if these events have increased during the recent period, our period of analysis was divided into two halves (as the period 1951–1983 (pre-1984), and 1984–2016 (post-1984)) each consisting of 33 years. Then, the average frequency of 1 and 3-day CHDHN events in these two periods was estimated (Figs 1 and S1), and the difference was examined (post-1984-pre-1984). We show that western, north-eastern, and southern part of India have experienced an increase of about three events (3-day CHDHN) per year in the post-1984 period. Furthermore, night-time extreme heat events are increasing more rapidly than daytime events (Fig. S2). The frequency of 3-day CHDHN events has declined over the Indo-Gangetic plain and part of eastern India in the post-1984 period (Fig. 1d). This decline in CHDHN events can be partially attributed to the influence of irrigation and atmospheric aerosols^21,22^.

The indo-Gangetic plain is one of the most heavily irrigated regions in the world^23^. Irrigation influences the surface energy budget over the region by increasing latent heat flux and decreasing sensible heat flux^24^. The increase in latent heat flux enhances evaporative cooling which in turn results in reduced surface air temperature^25,26^. A decreasing trend in pan evaporation in India has been reported^27^ during 1971–2010, which is mainly due to decline in short-wave radiation. However, both vegetation and evapotranspiration have substantially increased over the Indo-Gangetic Plain mainly due to intensive irrigation^21,28,29^. Therefore, increased evaporative cooling over the Indo-Gangetic Plain can be attributed to irrigation. Other than irrigation, atmospheric aerosols may also play a role in offsetting surface temperature by solar dimming over the Gangetic Plain, which has been reported in previous studies^30–33^. We confirmed this decline in the frequency of 3-day CHDHN over the Indo-Gangetic plain by estimating nonparametric (using Mann-Kendall test and Sen’s slope method) trends in 3-day mean daily average temperature for the 1951–2016 period (Fig. S3). A significant (p-value < 0.05) decline in 3-day mean temperature over the Indo-Gangetic Plain indicates the potential role of irrigation and atmospheric aerosols. Overall, the majority of western and southern India has experienced a significant increase in the frequency of 1 and 3-day CHDHN events during the post-1984 period (Figs 1d and S1d).

**Figure 2:**
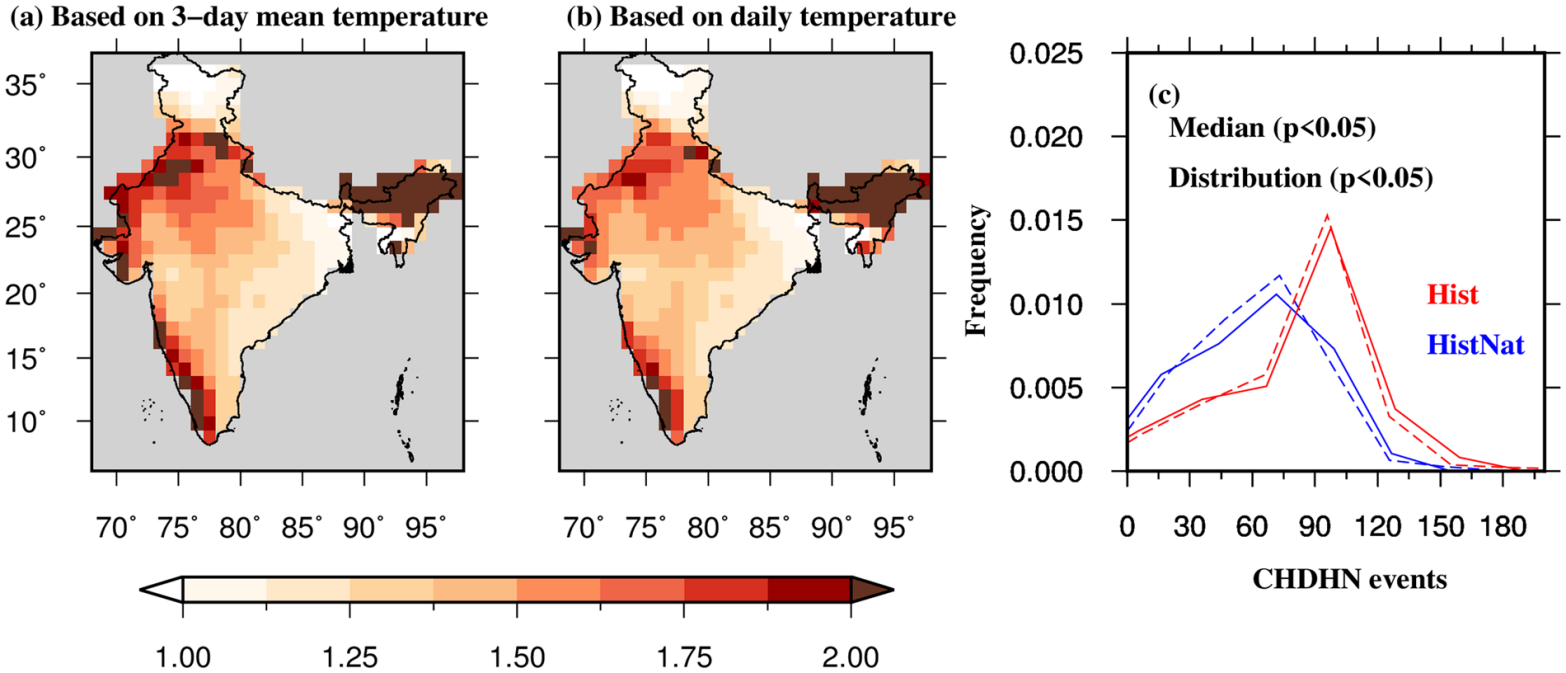
Influence of anthropogenic emissions on CHDHN events in India. (**a**) Multi-ensemble mean of ratio of 3-day CHDHN in the Hist to that in HistNat scenario during the period 1975–2013 based on 50 simulation from CAM5.1 model under the C20C+ D&A project. CHDHN ratio is estimated using 3-day moving mean daily temperature values, and (**b**) same as in (**a**) but for 1-day CHDHN ratio estimated using daily temperature data. (**c**) Empirical probability distribution of the number of CHDHN events in Hist (red), and HistNat (blue) scenarios based on 3-day mean moving mean daily temperature (solid line), and daily temperature (dashed line). The figure was developed using the Generic Mapping Tools (GMT, https://www.soest.hawaii.edu/gmt/).

While irrigation and atmospheric aerosols may alter the changes in CHDHN events locally^21,33^, anthropogenic warming can lead to a wide-spread and significant increase. To evaluate the role of anthropogenic emissions on the occurrence of the CHDHN events in India, we estimated the ratio of the number of CHDHN events based on the Hist and HistNat (Hist/HistNat) scenarios during 1975–2013 using 50 simulations from the C20C+ D&A project (see methods for details). The ratio is higher than 1 in the majority of India indicating that anthropogenic emissions contribute to the increased frequency of CHDHN events. Furthermore, western and southern India show that 1 and 3-day CHDHN events have increased 1.5–2 times while in northeastern India, these events have more than doubled due to anthropogenic emissions. Since the C20C+ simulations do not consider the influence of irrigation, the differences in the observed and C20C+ simulations over the Indo-Gangetic Plain are expected. The increase in 1 and 3-day CHDHN events in western and southern India is consistent with the observations (Figs 1 and S1). Overall, a statistically significant (p-value < 0.05) increase in 1 and 3-day CHDHN events is observed due to anthropogenic emissions over India (Fig. 2c).

**Figure 3:**
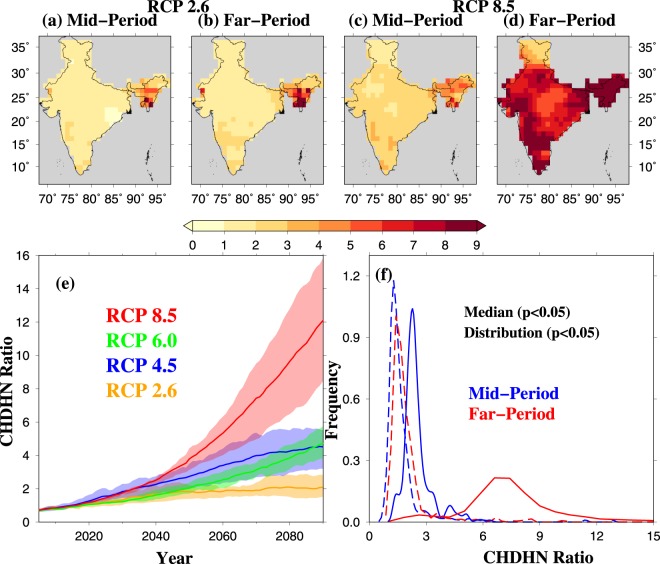
MME mean projected changes in 3-day CHDHN events in India. (**a**) 3-day CHDHN ratio for mid-period (2030–2050) based on RCP 2.6 emission scenario, (**b**) same as in (**a**) but for far-period (2070–2090), (**c**) same as in (**a**) but based on RCP 8.5 emission scenario, (**d**) same as in (**b**) but based on RCP 8.5 scenario, (**e**) all India averaged CHDHN ratio based on RCP 2.6 (orange), RCP 4.5 (blue), RCP 6.0 (green), and RCP 8.5 (red) during the period 2005–2090, and (**f**) Empirical probability distribution of CHDHN ratio based on RCP 2.6 (dashed-line) and RCP 8.5 (solid line) for mid-period (blue), and far-period (red). The CHDHN ratio is estimated based on 3-day moving mean of daily temperatures. The figure was developed using the Generic Mapping Tools (GMT, https://www.soest.hawaii.edu/gmt/).

Next, we analyze the temporal changes in 1 and 3-day CHDHN events in the projected future climate using data from eight CMIP5-GCMs for the four RCPs. For each 21-year moving window centered on each year from 2005 to 2090, the ratio (Future/Current) of the frequency of 1 and 3-day CHDHN events to the current world was estimated (21-year window centered on 2016 based on RCP8.5; see methods). Therefore, CHDHN ratio higher than one suggests an increase in the frequency of 1 and 3-day CHDHN events under the future climate. Under the RCP4.5 scenario, all India averaged frequency of 3-day CHDHN events (relative to the current world) is likely to become 4-fold by the end-21^st^ century (Figs 3e and S4e). If the global mean temperature continues to rise rapidly, India is projected to witness 4–5 fold rise in 3-day CHDHN events by mid-21^st^ century under RCP 8.5. More remarkably, India is projected to experience a 12-fold increase CHDHN events by the end of the 21^st^ century under RCP 8.5 (Figs 3e and S4e). This rise in 3-day CHDHN events under the projected warming will not be localized instead it is projected to cover a majority of India (Figs 3d and S4d). The benefit of climate change mitigation is well reflected as under the low-emission scenario (RCP 2.6), the increase in 3-day CHDHN events can be limited to only 2-fold by the end of the 21^st^ century in comparison to the 12-fold under RCP 8.5. The increase in both 1 and 3-day CHDHN events is consistent, and the projected future climate and is statistically significant at 5% level in the reference period (Figs 3f and S4f). To quantify the reliability of muli-model ensemble (MME) mean^34^, we estimated the ratio of MME mean and standard deviation (intermodel variation) for each RCPs for the projected future climate. Projections based on CMIP5 models showed the ratio (MME/std) greater than one indicating high reliability of CHDHN projections (Fig. S5b,d). We find that despite the intermodel variation among different RCPs (Fig. S5), our projections of 1 and 3-day CHDHN are robust (Fig. S5).

To compare the impact of low and high emission pathways (RCP 2.6 and RCP 8.5), we estimated changes in the frequency of 3-day CHDHN events in the mid (2030–2050) and far (2070–2090) periods to the current world (Figs 3a–d and S4a–d). This comparison between the two emission scenarios makes the impact of climate change more prominently visible and has been used in the previous study related to surface temperature over India^35^. The low emission scenario (RCP 2.6) is unlikely to lead to a substantial increase in 1 and 3-day CHDHN events in the mid and far periods of the 21^st^ century (Figs 3 and S4), which highlights the importance of the climate change mitigation. However, if the global mean temperature follows the high-emission pathway of RCP8.5, the frequency of 1 and 3-day CHDHN events is likely to become 3 and 8-fold of the current climate in the mid and far periods (Figs 3 and S4). In addition to that, the increase in the frequency of 1 and 3-day CHDHN events in the mid and far periods are found to be statistically significant at 5% level based on the RCP 8.5 scenarios (Figs 3f and S4f). Under the two intermediate scenarios of RCP 4.5, and 6.0, the frequency of 1 and 3 day CHDHN events is projected to increase by 4–6 fold during the far-period (2070–2090) (Fig. S6), which is higher than the low emission scenario of RCP 2.6. Our results again highlight the benefits of climate change mitigation as the projected increase in the low-emission scenario (RCP 2.6) is much lesser than the other emission scenarios (RCP 4.5, 6.0, and 8.5).

**Figure 4:**
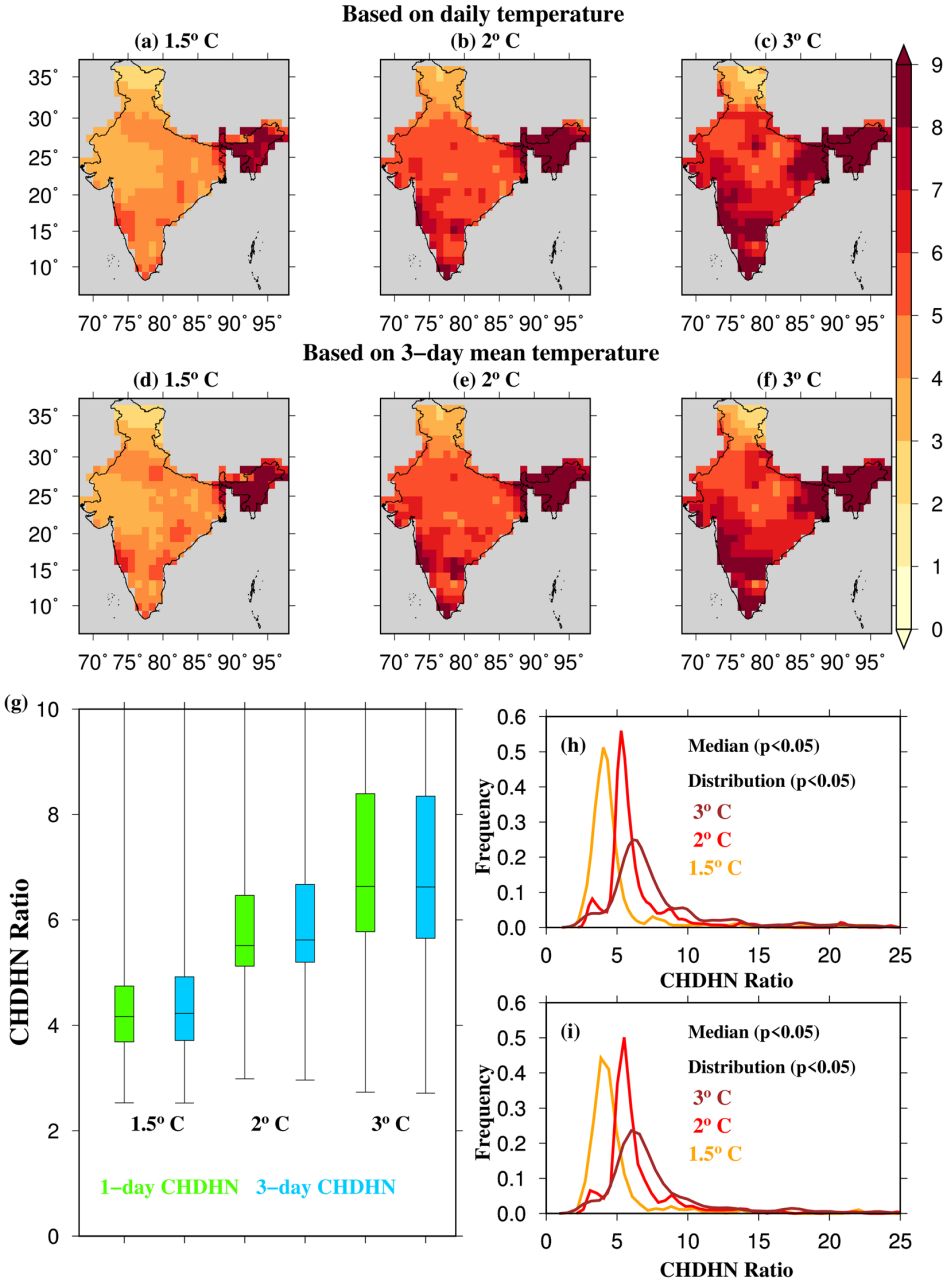
Projected changes in 1 and 3-day CHDHN events under 1.5, 2, and 3.0 °C warming worlds. (**a**) The ratio of number of 1-day CHDHN events in the 1.5 °C warming world to that in the current period (2006–2026 of RCP8.5 scenario; CHDHN ratio), (**b** and **c**) same as in (**a**) but for 2 °C, and 3 °C warming worlds, respectively, (**d**–**f**) same as in (**a**–**c**) but for 3-day CHDHN ratio (**g**) Box and whisker plot showing median, and interquartile range for 1 and 3-day CHDHN ratio. (**h**) Empirical probability distribution of 1-day CHDHN ratio, and (**i**) same as in (**k**) but for 3-day CHDHN ratio. The figure was developed using the Generic Mapping Tools (GMT, https://www.soest.hawaii.edu/gmt/).

Finally, we estimated the changes in the frequency of 1 and 3-day CHDHN events under the 1.5, 2 and 3 °C warming worlds. The Paris agreement aims to limit the global mean temperature below 2 °C and more ambitiously below 1.5 °C from the pre-industrial level by the end of 21^st^ century. Therefore, changes in the frequency of 1 and 3-day CHDHN events under these temperature targets were estimated to determine the potential benefits of climate change mitigation. Additionally, we considered the 3 °C warming world for our analysis. Raftery *et al*.^36^ argued that the global mean temperature is most likely to overshoot the 1.5 and 2 °C limits and reach the 3 °C (ranging between 2 °C to 4.9 °C) limit by the end of 21^st^ century. The ratio of the number of 1 and 3-day CHDHN events under the 1.5, 2, and 3 °C warming worlds to the number of 1 and 3-day CHDHN events was estimated under the current climate (Fig. 4). One and 3-day CHDHN events are projected to increase by 4, 6, and 8-folds (of the current level) in India under the 1.5, 2, and 3 °C warming worlds, respectively (Fig. 4). Moreover, all India averaged frequency of 1 and 3-day CHDHN events (Fig. 4g) is likely to increase by 4, 5.5, and 6.5 fold increase under the 1.5, 2, and 3 °C temperature targets respectively. The projected increase in 1 and 3-day CHDHN events under the different temperature targets is statistically significant at 5% level for both mean and distribution (Fig. 4).

The CHDHN events are likely to rise substantially in India under the warming climate. Change in mean and distribution of summer temperature under the future climate can result in an increased frequency of CHDHN events under different (1.5–3.0 °C) warming worlds. Increased frequency of heatwaves is more prominently linked with the change in mean rather than the change in the distribution of temperature^4^. However, other dynamical features and large-scale teleconnections can influence the frequency of CHDHN events under the current and projected future climate^9,10,37–39^. For instance, heatwaves over north India are linked with blocking of north Atlantic, which results in a cyclonic anomaly over the west of North Africa^9^. Ratnam *et al*.^9^ reported that anomalous cooling over the Pacific causes the occurrence of heat waves over coastal eastern India. Heatwaves in India are influenced by not only the sea surface temperature (SST) anomalies over the Pacific but also by the SST conditions in the Tropical Indian Ocean^18^. Under the warming climate, the frequency of El Nino is projected to rise^40,41^, which can result in more temperature extremes over India^18^.

Other than the large-scale climate features (e.g., ENSO), the frequency of CHDHN events in India under the current and projected future climate can be affected by local conditions (land use/land cover, irrigation, and aerosols) related to land surface and atmosphere. For instance, intensive irrigation and aerosols over the Indo-Gangetic Plain can reduce surface and air temperature^21,22^. Presence of aerosols can influence solar radiation^30^, which in turn can result in changes in the diurnal temperature range (DTR). Our analysis show that CMIP5 models capture the observed DTR variability during the summer season reasonably well (Fig. S7a,b) with low intermodel variation in the majority of India (Fig. S7c). Additionally, other than local and large-scale factors, a combination of high temperature and humidity can cause heat stress^6^, however, our aim was to estimate the changes in CHDHN events based on air temperature in the future climate. Our estimates of reliability (Fig. S7d) confirm the robust increase in CHDHN events in India under the warming climate^42^. Understanding the role of local factors and large-scale teleconnections that can provide the physical explanation of temperature extremes over India is essential and can be attempted in future studies.

## Conclusions

We provided the first-ever assessment of concurrent heatwaves in India with the following primary conclusions:

1. The frequency of 1 and 3-day CHDHN events has increased in large part of western and southern India during the post-1984 period. Night-time heat events have increased more rapidly than the day-time heat events during the recent few decades in India. However, Indo-Gangetic Plain and eastern parts have experienced a decline in the frequency of 1 and 3-day CHDHN events, which can be partially associated with the local cooling due to irrigation and atmospheric aerosols.
2. The frequency of 1 and 3-day CHDHN events increases significantly under the anthropogenic emissions. Under the high emission scenario of RCP 8.5, the frequency of 3-day CHDHN events is projected to increase 12-fold of the current level by the end of 21^st^ century and 4-fold by the mid 21^st^ century. On the other hand, the frequency of 3-day CHDHN is projected to increase to 4 and 2-fold of the current frequency by the end of the 21^st^ century under the emission scenarios of RCP 4.5 and 2.6. The difference in the rise in the frequency of CHDHN events in India under the high and low emission scenarios highlights the importance of climate change mitigation.
3. Limiting global mean temperature to 1.5 °C from the pre-industrial level can be beneficial in reducing the risks of 1 and 3-day CHDHN events in India. For instance, a rise of 1.5 °C in the global mean temperature is projected to cause a 4-fold increase in the frequency of 3-day CHDHN from the current level. An additional warming of 0.5 °C (under 2 °C warming world) is likely to cause an increase of 6-fold in the frequency of 3-day CHDHN events in India. Moreover, failing to limit the global mean temperature below 2 °C can lead to a rise in 8-fold in the frequency of 3-day CHDHN events in India (under 3 °C target).
4. The significant rise in the 1 and 3-day CHDHN events in India under the projected future climate may pose severe implications for public health and heat-related mortality in India^8,43^.

## Data and Methods

We used the daily maximum (Tmax) and minimum (Tmin) air temperature for the period 1951–2016 from the India Meteorological Department (IMD) to estimate CHDHN events in the observed climate. The gridded temperature data are developed using Shepard’s distance weighted interpolation^44^ using data from 395 observational stations located across India, which are available at 0.5° spatial resolution. We have regridded the temperature data to 1° spatial resolution using bi-linear interpolation to make it consistent with the data from Coupled Model Intercomparison Project Phase 5 (CMIP5)^45^ and CAM5.1 simulations under the C20C+ (Folland *et al*.^46^) D&A project.

To investigate the role of anthropogenic emission on CHDHN events, we obtained daily Tmax and Tmin data at the 1° spatial resolution for the Hist and HistNat scenarios from 50 simulations of the CAM5.1 model under C20C+^46^ D&A project for 1975–2013. The C20C+ project is a part of the World Climate Research Programme’s (WCRP) International Climate and Ocean-Variability, Predictability, and Change (CLIVAR) 5^th^ workshop, held in Beijing in October 2010. The main purpose of the C20C+ project is to measure what levels the extreme weather events are attributed to anthropogenic warming.

For the projected future climate, we obtained daily Tmax and Tmin data from eight GCMs that participated in the CMIP5^45^. We selected the GCMs based on their availability for the historical scenario and the four representative concentration pathways (RCPs: RCP2.6, RCP4.5, RCP6.0, and RCP8.5) for the r1i1p1 realization available from the period 1950 onwards. The four RCPs correspond to the four different levels of emission scenarios for the future climate with RCP8.5 representing the highest and RCP2.6 representing the lowest emission scenarios. The data were regridded to 1° spatial resolution using bi-linear interpolation to make it consistent with the other (IMD and C20C+) datasets. The regridded temperature was then compared against temperature at GCMs’ native resolution, and we found that the interpolated data were consistent spatially and temporally.

The selected GCMs show both negative and positive bias in Tmax and Tmin against the observed data (Fig. S1). Warm bias in CMIP5 GCMs is centered mainly in the central and western India (Fig. S8). Furthermore, different GCMs may have different land surface conditions, which can affect surface temperature simulations. For instance, the surface temperature gets amplified if the interaction between land surface and clouds are not well represented in the models^47^ and may result in an over or underestimation of GCMs simulated evapotranspiration^48^. To understand uncertainty CHDHN events, we estimated multi-model mean and standard deviation (uncertainty) for diurnal temperature range (DTR) during the summer season (AMJ) for the historic period of 1971–2000 (used as reference period to estimate CHDHN events). Consistent with the warm bias over the western and central India, we notice an intermodel uncertainty of 1–3 °C in DTR inCMIP5-GCMs (Fig. S7c).

A percentile-based approach was used to estimate the CHDHN events based on the summer (April to June) daily Tmax and Tmin during the reference period of 1971–2000. Hot days (nights) for each grid location are identified if Tmax (Tmin) exceeds the 95^th^ percentile threshold for the reference period (1971–2000). Then CHDHN events were identified if a hot day and hot night occur for the same day for a given period (Fig. S9). Lin *et al*.^49^ and Karl and Night^17^ reported that extreme temperature events occurring continuously for 3 or more days pose a significant threat to human health. Therefore, we analyze the CHDHN events following the same methodology (as for daily events) but using the 3-day moving mean of daily Tmax and Tmin. Thus, our analysis is based on two sets of CHDHN events, one based on the daily Tmax and Tmin data [1-day CHDHN] and the other is based on the 3-day Tmax and Tmin [3-day CHDHN].

Extreme climatic events attributed to increased greenhouse gas emission have significantly increased notably since the mid-1970s^50^. Therefore, we focus on the period 1975 onwards to study the role of anthropogenic emission on CHDHN events. To do so, we estimated the ratio of the number of CHDHN events in the Hist to the number of CHDHN events in the HistNat scenario. Since the Hist scenario incorporates the anthropogenic forcings unlike the HistNat scenario which represents the climate without human influence, a ratio higher than one indicates the presence of the anthropogenic contribution to the CHDHN events.

The changes in the frequency of the CHDHN events under the projected future climate were estimated based on the 8 CMIP5-GCMs for the four RCPs to the current world. We define the current world as the period of 21 years centered on 2016 based on the highest emission scenario (RCP8.5) to avoid the overestimation in our estimates as described in King *et al*.^51^. This is also justified as the observed increase in the global mean temperature is higher than the lower emission scenarios^36^. Temporal change in CHDHN events throughout the 21^st^ century was analysed based on the ratio of the frequency of CHDHN events (CHDHN ratio) in the 21 year moving window, centered on each year from 2005 to 2090 to the frequency of CHDHN events in the current world (RCP 8.5 scenario for the 21 year window centered on 2016).

Finally, the changes in CHDHN events were analysed under the warming limits of 1.5, 2, and 3 °C rise in global mean temperature to the pre-industrial period (1861–1900). We followed the same procedure as used in King, *et al*.^51^ and selected the model years corresponding to each warming limit (1.5, 2, and 3 °C) and refer them as 1.5, 2, and 3 warming world. First, we estimated temperature anomaly of the decadal average of global mean temperature using the baseline period (pre-industrial:1861–1890) under the historical scenario. We selected the 1.5 °C world for each model as all years within the decades with temperature 1.3–1.7 °C warmer than the corresponding model baseline for all the RCPs. For the 2 °C and 3 °C warming worlds, the same method was applied, only the temperature range was changed to 1.8–2.2 °C, and 2.8–3.2 °C, respectively. Then, the CHDHN ratio was estimated for each grid as the ratio of the number of CHDHN events in the 1.5, 2, and 3 °C worlds, respectively, to that in the current world.

## Electronic supplementary material

Supplementary Information

## Data Availability

All the data used in this study will be made available on request to the corresponding author.
