# Supplementary material for: A sixfold rise in concurrent day and night-time heatwaves in India under 2 °C warming
Source: Sci Rep. 2018 Nov 16;8:16922. doi: 10.1038/s41598-018-35348-w (PMC6240077; doi:10.1038/s41598-018-35348-w)
Supplement: Supplementary file 1 — Supplementary Information [file 41598_2018_35348_MOESM1_ESM.docx]

**Supplemental Information**

**A sixfold rise in concurrent day and night-time heatwaves in India under 2ºC warming**

Sourav Mukherjee and Vimal Mishra*

Civil Engineering, Indian Institute of Technology Gandhinagar, Gujarat, India, 382355

Corresponding Author: vmishra@iitgn.ac.in


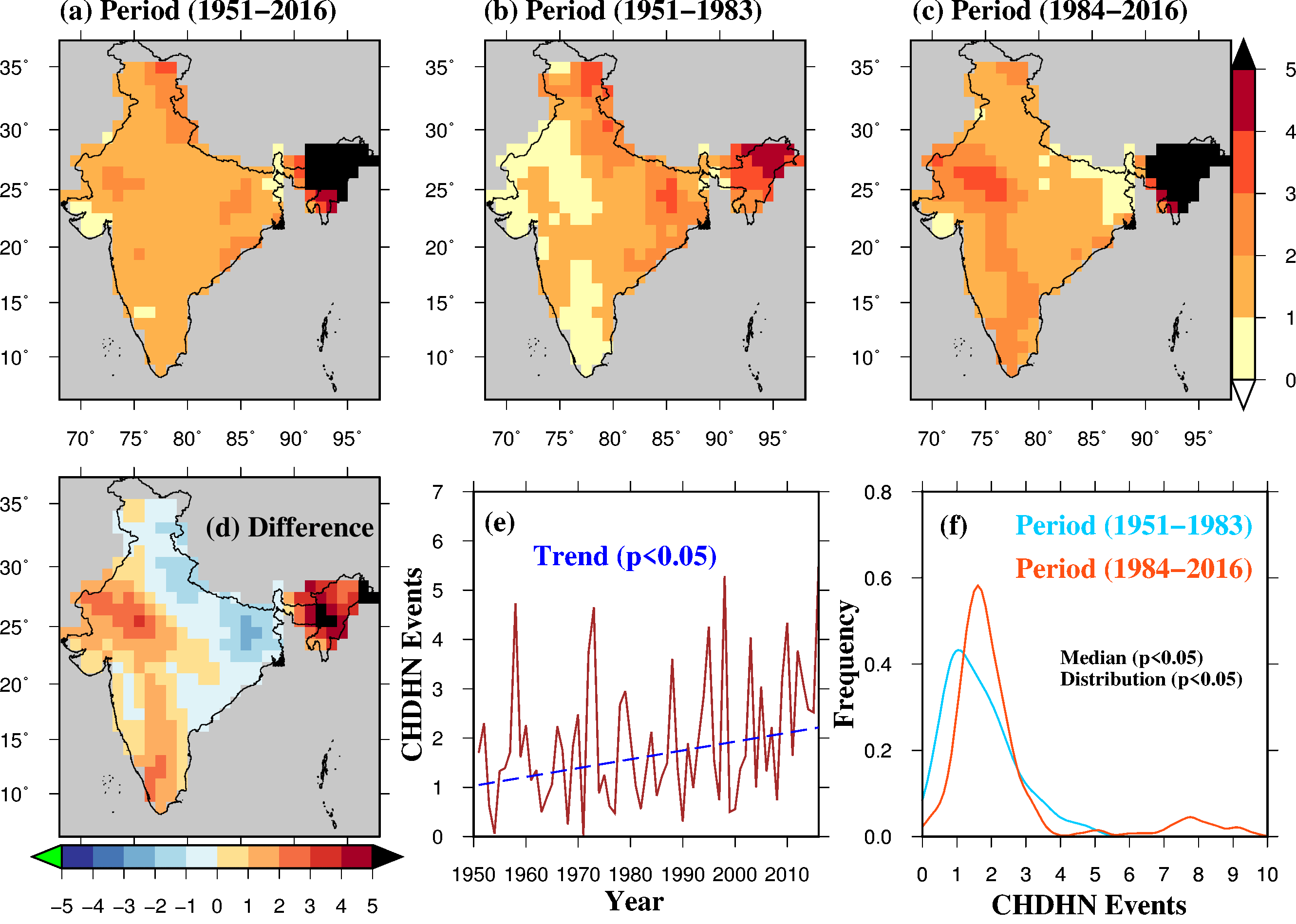


Figure S1 same as in Figure 1 but for1-day CHDHN events. The figure was developed using the Generic Mapping Tools (GMT, https://www.soest.hawaii.edu/gmt/).

**
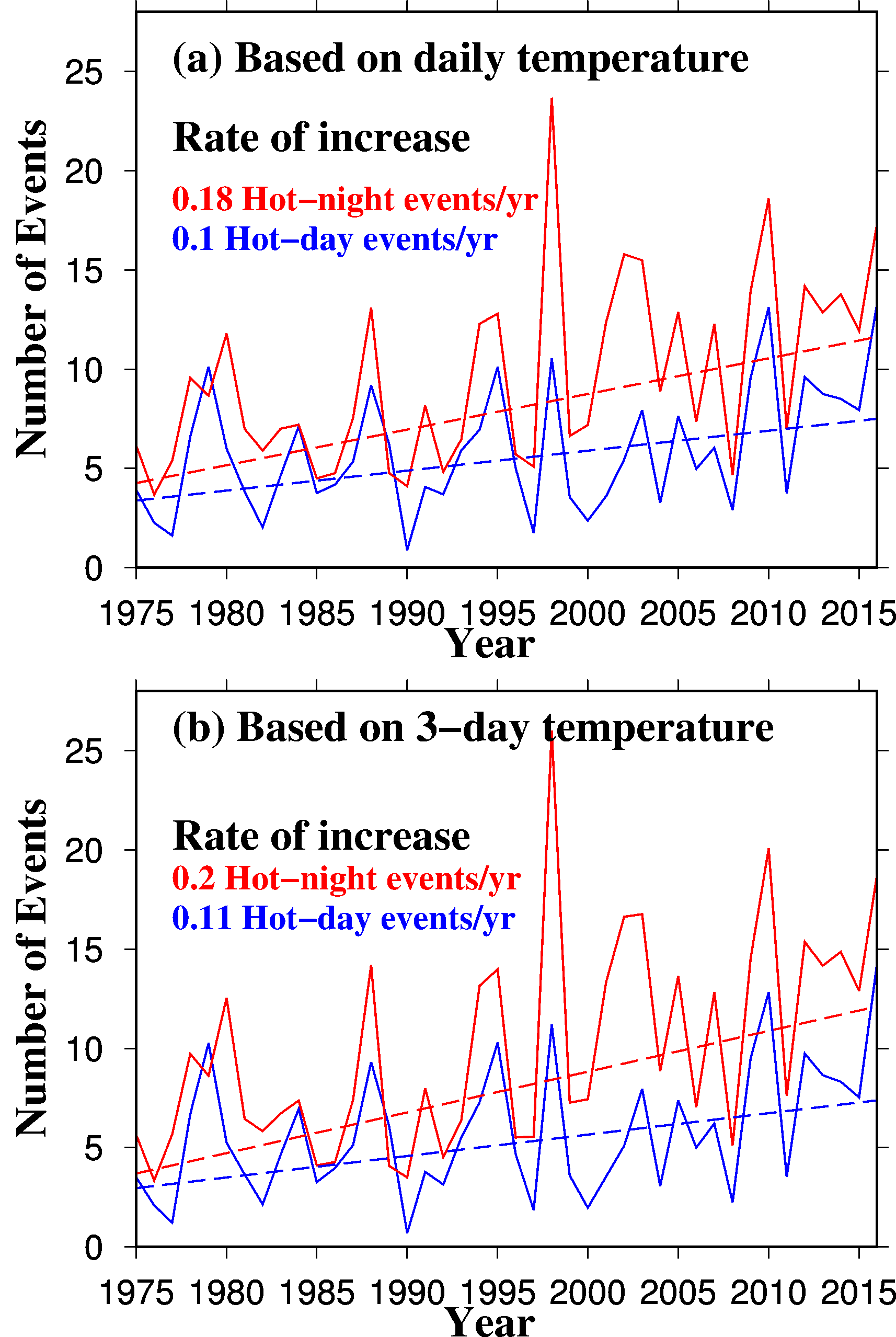
**

Figure S2 (a) All India averaged time series for Number of hot-night (red) and hot-day (blue) events based on daily temperature data, (b) same as in (a) but based on 3-day moving mean od daily temperature data. The dashed lines showing the trend in the number of hot-night (red) and hot-day (blue) events estimated at 5% significance level. The figure was developed using the Generic Mapping Tools (GMT, https://www.soest.hawaii.edu/gmt/).


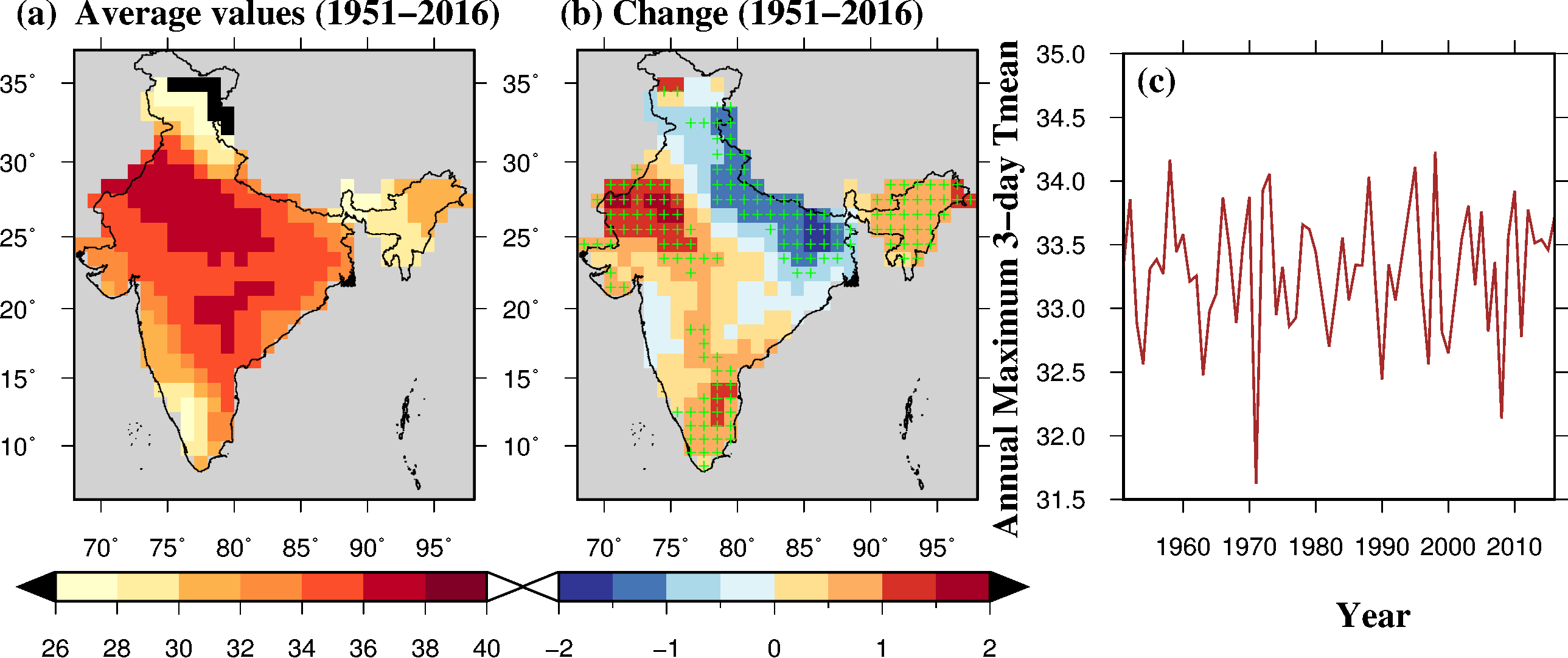


Figure S3 (a) Mean of annual maximum 3-day mean of daily average temperature during the climatological period, 1951-2016. (b) Change in the annual maximum 3-day mean of daily average temperature during the same period, and (c) all India average of the annual maximum 3-day mean of daily average temperature during the same period. The figure was developed using the Generic Mapping Tools (GMT, https://www.soest.hawaii.edu/gmt/).


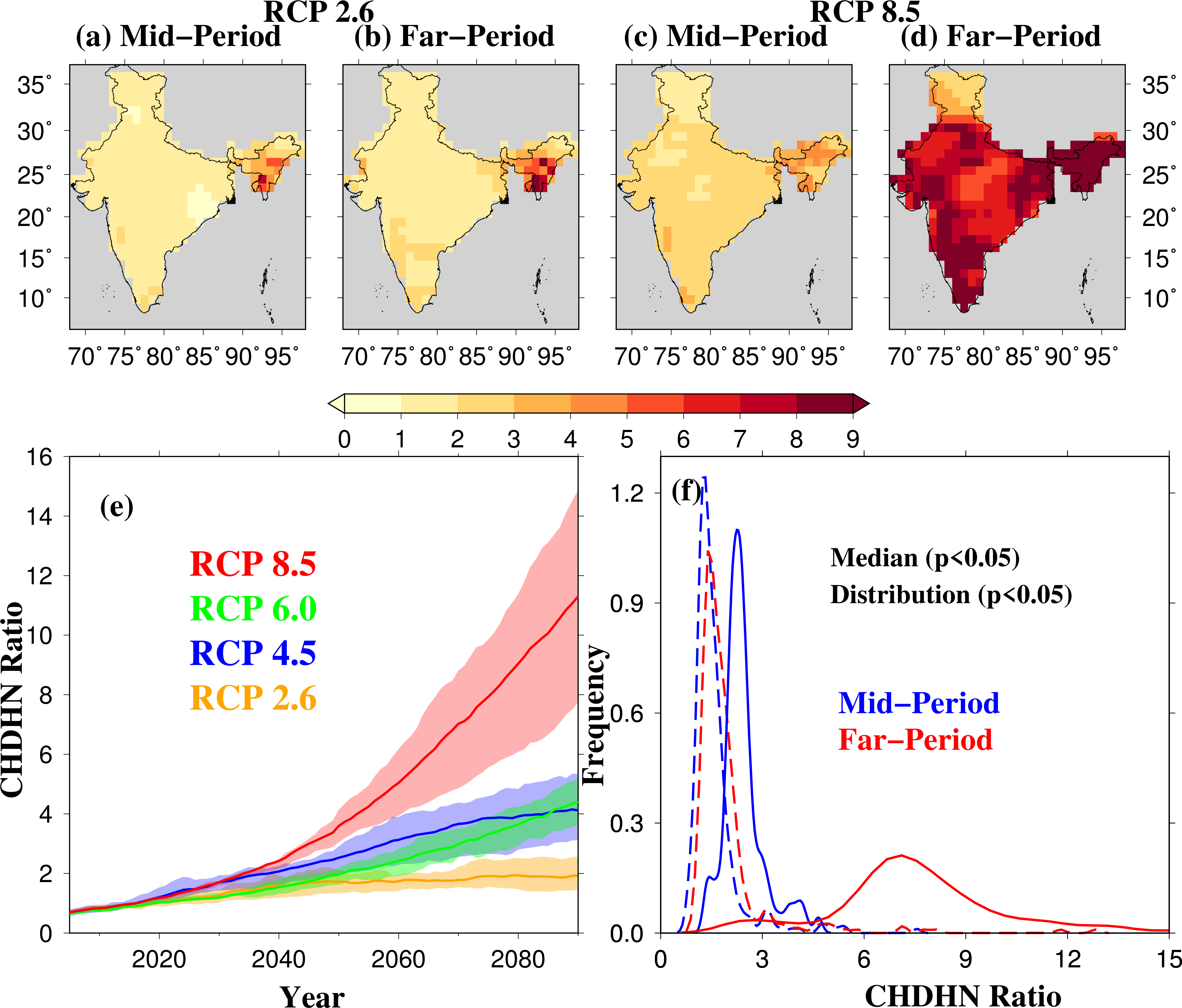


Figure S4 Same as in Figure 3 but CHDHN ratio estimated using daily temperatures. The figure was developed using the Generic Mapping Tools (GMT, <https://www.soest.hawaii.edu/gmt/>).


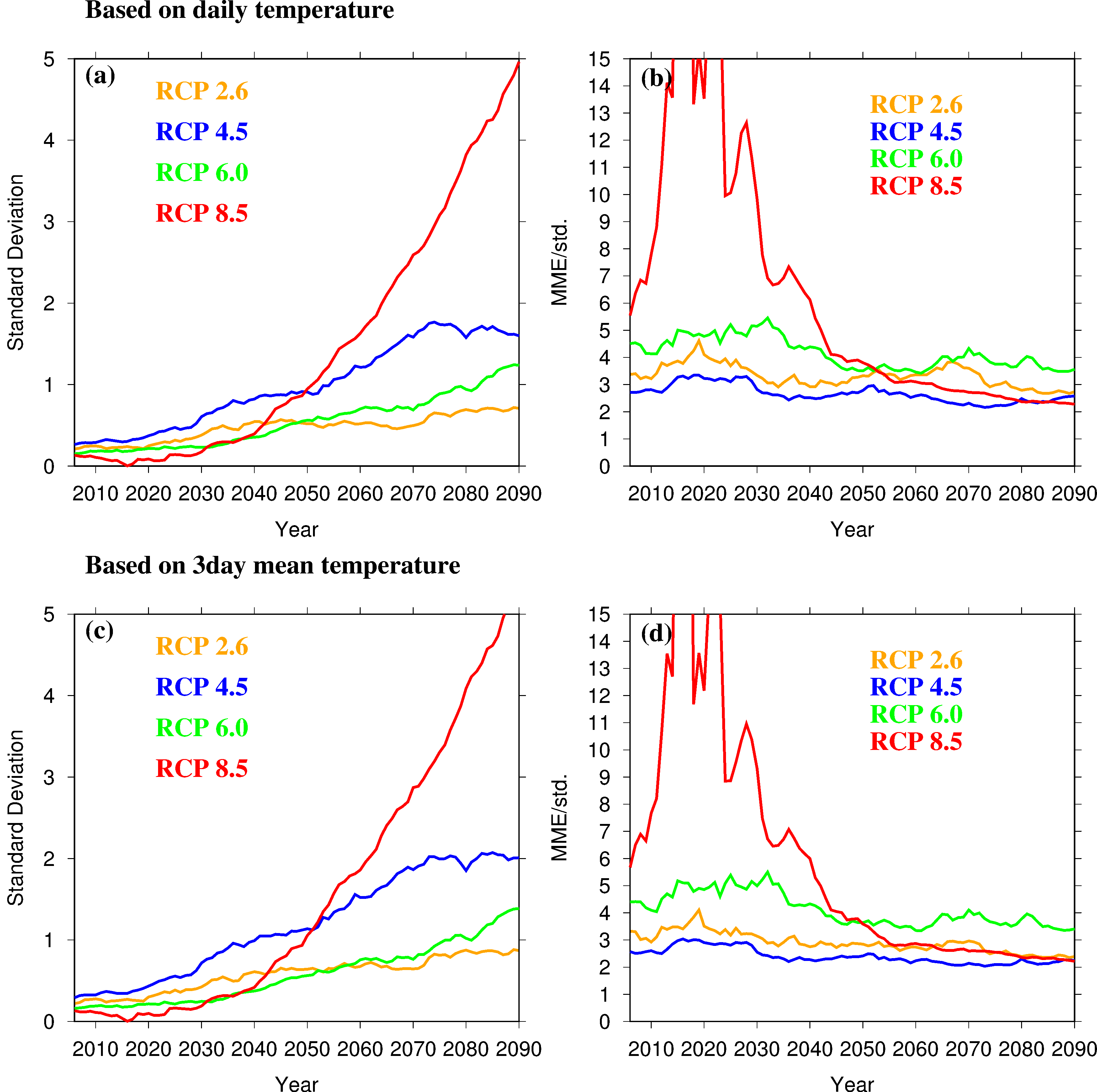


Figure S5 (a) Uncertainty of CHDHN ratio estimated based on 8 CMIP5-GCMs based on daily temperature data, (b) ratio of MME mean to the standard deviation for CHDHN ratio from 8-CMIP5-GCMs based on daily temperature data, (c) same as in (a) but for CHDHN ratio estimated based on 3-day mean temperature, and (d) same as in (b) but for CHDHN ratio estimated based on 3-day mean temperature. The figure was developed using the Generic Mapping Tools (GMT, <https://www.soest.hawaii.edu/gmt/>).


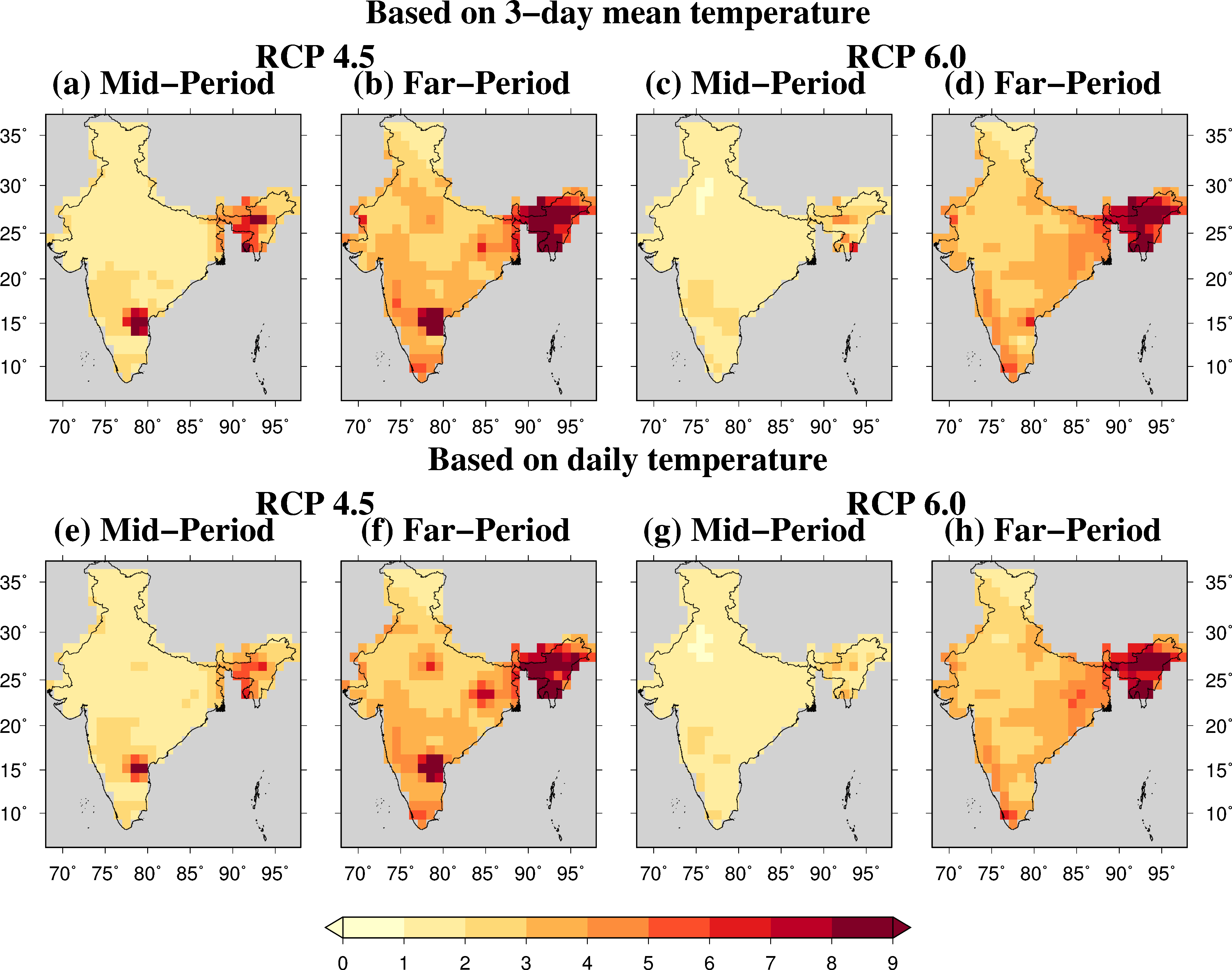


Figure S6 **MME mean projected changes in 3-day CHDHN events in India.** (a) 3-day CHDHN ratio for mid-period (2030-2050) based on RCP 4.5 emission scenario, (b) same as in (a) but for far-period (2070-2090), (c) same as in (a) but based on RCP 6.0 emission scenario, (d) same as in (b) but based on RCP 8.5 scenario, (e-h) same as in (a-d) but based on daily temperature. The figure was developed using the Generic Mapping Tools (GMT, <https://www.soest.hawaii.edu/gmt/>).


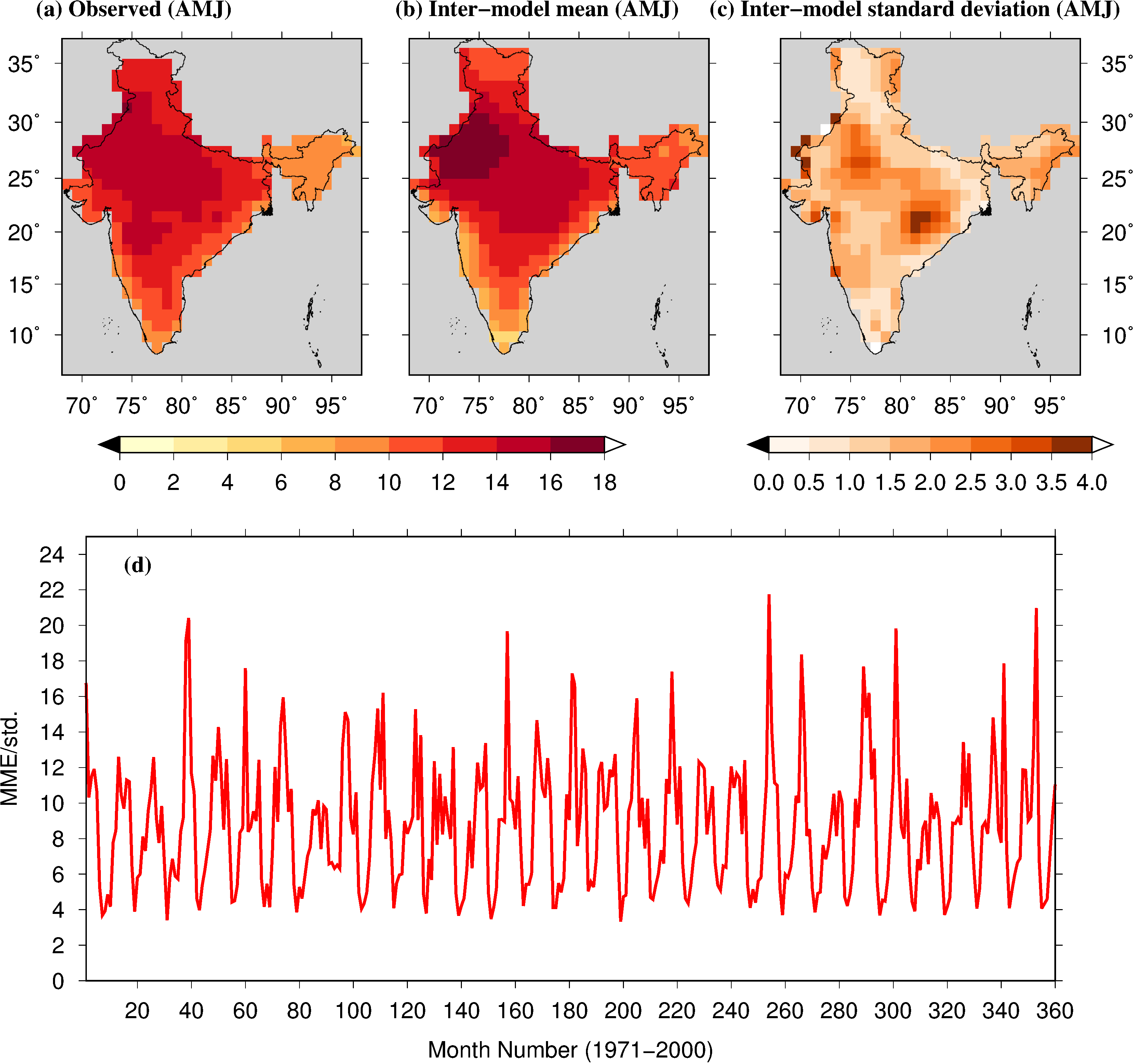


Figure S7. Spatial distribution of (a) Diurnal temperature change for the summer season (AMJ) during the period 1971-2000 based on observed dataset. (b) Inter-model mean of diurnal temperature change for the summer season (AMJ) during the period 1971-2000. (c) Inter-model standard deviation of diurnal temperature change for the summer season (AMJ) during the period 1971-2000. (d) Reliability (=MME/std.) of CMIP5-GCMs to capture the seasonal cycle of diurnal temperature change during the period 1971-2000. The figure was developed using the Generic Mapping Tools (GMT, <https://www.soest.hawaii.edu/gmt/>).


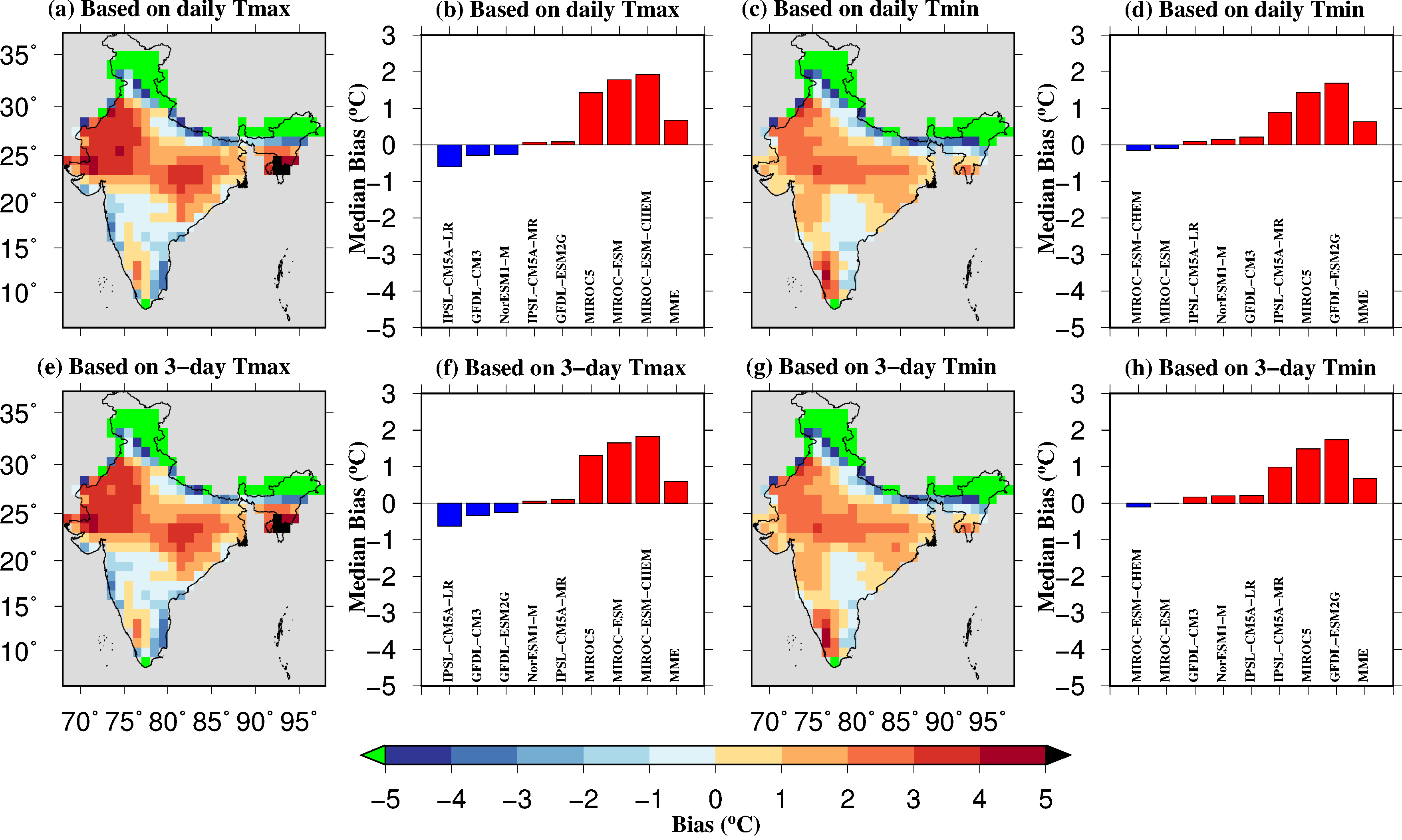


Figure S8. Spatial distribution of MME mean bias against observation in 95^th^ percentile of (a) daily Tmax, (c) daily Tmin, (e) 3-day moving mean of daily Tmax, and (g) 3-day moving mean of daily Tmin during the climatological period 1971-2000, (b, d, f, and h) all-India median bias against observation for 8 individual CMIP5-GCMs and their MME mean during the period 1971-2000 based on daily Tmax, daily Tmin, 3-day moving mean of daily Tmax, and 3-day moving mean of daily Tmin, respectively. The figure was developed using the Generic Mapping Tools (GMT, <https://www.soest.hawaii.edu/gmt/>).

**
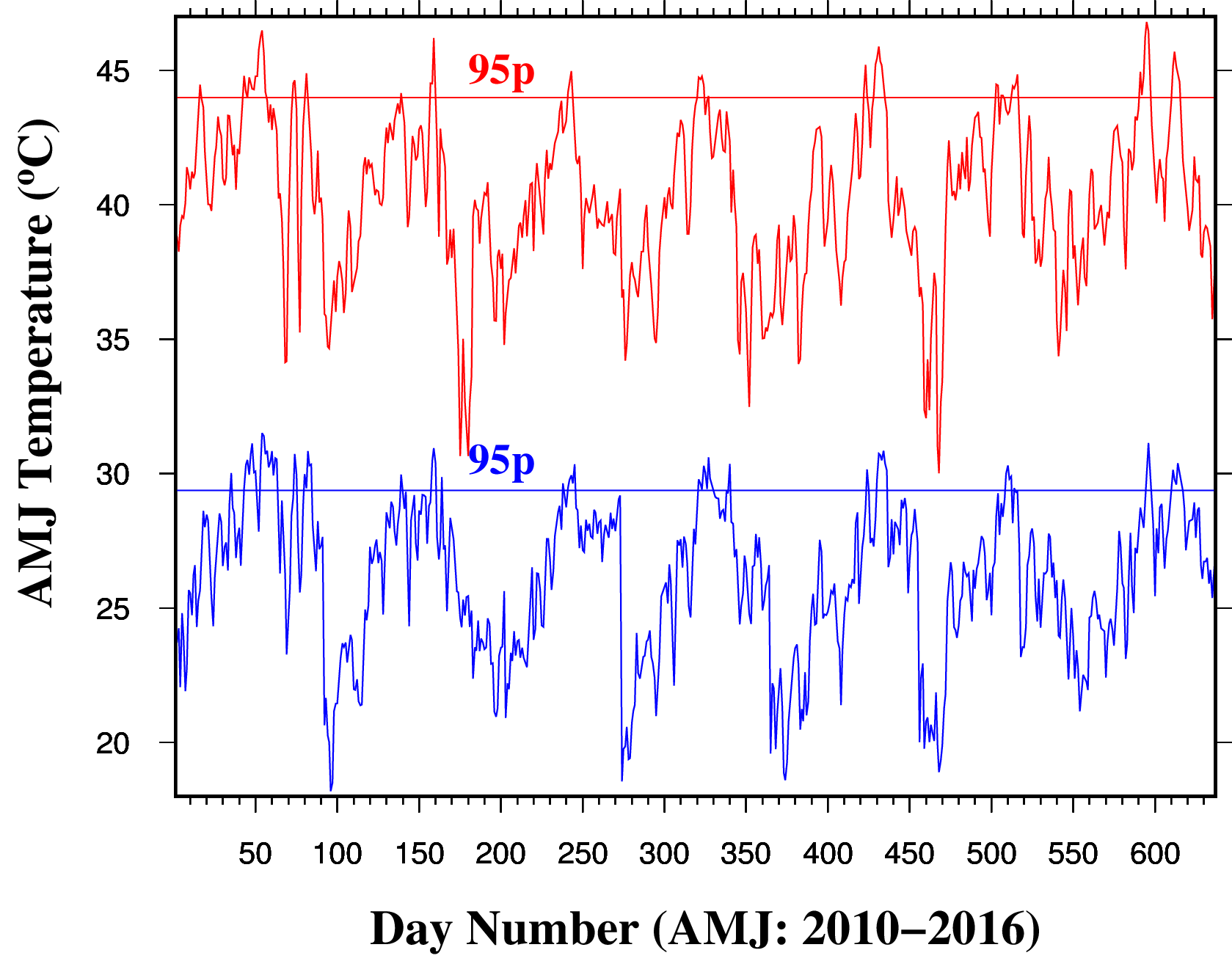
**

**Figure S9.** Daily summer (AMJ) Tmax (red) and Tmin (blue) distribution for the days corresponding to the summer season during 2010-2016. The horizontal lines represent 95^th^ percentile of AMJ daily Tmax (red) and daily Tmin (blue) during the climatological period 1971-2000. The figure was developed using the Generic Mapping Tools (GMT, https://www.soest.hawaii.edu/gmt/).

Table S1: List of 8 CMIP5-GCMs (Table S1) at 1º spatial resolution used based on the historical and RCPs (RCP 2.6, RCP 4.5, RCP 6.0, and RCP 8.5) scenarios

| Sl. No. | Model | Historical | RCP 2.6 | RCP 4.5 | RCP 6.0 | RCP 8.5 |
| --- | --- | --- | --- | --- | --- | --- |
| 1 | GFDL-CM3 | 1900-2005 | 2006-2100 | 2006-2100 | 2006-2100 | 2006-2100 |
| 2 | GFDL-ESM2G | 1861-2005 | 2006-2100 | 2006-2100 | 2006-2100 | 2006-2100 |
| 3 | IPSL-CM5A-LR | 1850-2005 | 2006-2100 | 2006-2100 | 2006-2100 | 2006-2100 |
| 4 | IPSL-CM5A-MR | 1850-2005 | 2006-2100 | 2006-2100 | 2006-2100 | 2006-2100 |
| 5 | MIROC-ESMC | 1850-2005 | 2006-2100 | 2006-2100 | 2006-2100 | 2006-2100 |
| 6 | MIROC-ESM-CHEM | 1850-2005 | 2006-2100 | 2006-2100 | 2006-2100 | 2006-2100 |
| 7 | MIROC5 | 1850-2005 | 2006-2100 | 2006-2100 | 2006-2100 | 2006-2100 |
| 8 | NorESM-1M | 1900-2005 | 2006-2100 | 2006-2100 | 2006-2100 | 2006-2100 |
